# Recognition without authority: Cormac McCarthy’s Stella Maris and the gender visibility debate

**DOI:** 10.3389/fsoc.2026.1956261

**Published:** 2026-09-23

**Authors:** Madina Radjabova

**Affiliations:** Department of English Literature and Translation Studies, Bukhara State University, Bukhara, Uzbekistan

**Keywords:** gender visibility, institutional recognition, intersectionality, non-performativity, symbolic representation, tokenism

## Abstract

Gender visibility days aim to mark and correct the underrepresentation of women in specific institutions and fields. This article argues that the empowerment-versus-ritual debate in gender visibility scholarship is missing a conceptual distinction already present in the sociology of tokenism and institutional diversity work: the difference between being recognized and being granted authority. Drawing on sociological theories of tokenism, institutional non-performativity, and intersectionality, this article proposes “recognition without authority” as an analytic category for evaluating gender-focused observances. It illustrates the category through a single case: Cormac McCarthy’s novel Stella Maris (2022), whose protagonist is watched, examined, and discussed at length within institutions dominated by men, yet whose testimony is never treated as credible on its own terms. The case serves as a practical example of a dynamic that future sociological research on visibility days can test more systematically across different institutional settings.

## Introduction

Gender-focused observances, from International Women’s Day to the International Day of Women and Girls in Science, have proliferated against a backdrop of persistent global inequality. The World Economic Forum’s 2025 Global Gender Gap Report finds that only 68.8% of the global gender gap has closed, with full parity projected 123 years away at current rates (World Economic Forum, 2025). Against this backdrop, a recurring question in the sociology of gender observances is whether these days function as empowerment or settle into routine ritual: does marking an issue once a year translate into institutional change, or does it substitute for it? Empirical work on the largest of these observances suggests the answer is not straightforward: Zumeta et al. (2020), in a cross-cultural study of over 2,800 demonstrators and non-demonstrators, found that participation in 8 March/International Women’s Day demonstrations was associated with heightened well-being, solidarity, and collective efficacy—real effects, but ones that speak to the experience of participants rather than to downstream institutional change, which is the question this article takes up.

Much of this debate implicitly treats visibility as a resource that should, in principle, convert into structural change. This article argues that the debate needs a more precise conceptual tool than “visibility” alone can supply. Sociological work on tokenism and institutional diversity work already distinguishes between being seen and being empowered. By introducing the concept of recognition without authority into gender visibility studies, this article argues that separating representation from influence provides a more precise analytical framework, as illustrated through a single case study.

## Tokenism: visibility as exposure, not power

Kanter (1977) foundational theory of tokenism describes how individuals who are numerically rare within an organization become hypervisible. As a result, they face intense scrutiny, symbolic representation of their category, and pressure to conform—all without gaining influence over how they are judged. Tokenism theory’s central insight is precisely the one gender visibility scholarship needs: visibility and power are not the same axis and increasing one does not reliably increase the other. A token is watched closely and discussed extensively, and this scrutiny can intensify rather than relieve institutional pressure, because the token’s every action is read as representative of a category rather than as an individual judgment. This dynamic is not merely historical: Chang et al. (2019) find that U.S. corporate boards are significantly more likely to include exactly two women than chance alone would predict—a pattern they term “twokenism”—and that this pattern is more pronounced among more visible companies, consistent with impression-management motives rather than any corresponding increase in women’s decision-making authority.

This reframes what a gender visibility day can produce. If tokenism theory correctly separates hypervisibility from institutional power, then an annual visibility observance is inherently limited. It addresses only a single factor in whether marginalized groups can build lasting institutional influence.

## Non-performativity: when institutional recognition substitutes for change

Ahmed (2006) concept of institutional non-performativity extends this point from individuals to institutions themselves. Ahmed highlights how institutional commitments to equality can act as non-performatives. Declaring a commitment does not trigger real change, and it often creates the illusion that the institution has already fulfilled its obligation. Research on gender visibility days often distinguishes between grassroots activism and institutional adoption (de Jong and Kimm, 2017), noting that once an observance is absorbed into a corporate or academic calendar, its critical edge tends to dull (see also Banet-Weiser, 2018, on the broader phenomenon of feminist visibility becoming a commodified end in itself rather than a means to structural change). Ahmed’s framework gives this dulling a precise mechanism: the observance itself becomes the institution’s evidence of having “done” gender equity, foreclosing further pressure for structural change. This mechanism is visible in corporate practice: Walters (2022) analyzes transnational corporations’ gender-focused corporate social responsibility programs as a form of “gender wash,” in which visible investment in women’s empowerment messaging coexists with continued gendered inequities in the same firms’ supply chains and employment practices—an empirical illustration of the gap between declared commitment and structural change that non-performativity theory predicts.

## Intersectionality: the limits of a single-axis observance

A further limitation of single-day observances stems from Crenshaw (1989) foundational argument on single-axis discrimination. Frameworks built around one category fail individuals whose marginalization arises from the intersection of several, such as gender combined with disability, mental health status, race, or class. An observance organized around a single identity category cannot, by design, represent a subject whose exclusion is intersectional rather than singular. This limitation is not hypothetical. For a single-day observance to succeed as a public ritual, it needs an easily recognizable, single-category focus that can be readily celebrated, which structurally pushes intersectional cases like Alicia’s, discussed below, outside what the ritual can register.

## An illustrative case: institutional recognition without authority

The disconnect between recognition and authority is clearly illustrated by Alicia Western, the protagonist of Cormac McCarthy's Stella Maris (McCarthy, 2022). Her character provides a concrete example of how visibility can exist entirely apart from institutional power. The novel is set in 1972 and takes the form of transcribed sessions between Alicia, a twenty-year-old mathematician, and her psychiatrist at a Wisconsin sanatorium, where she has voluntarily admitted herself. Alicia’s institutional recognition is, by any ordinary measure, extensive: she was identified as intellectually exceptional in early childhood, entered a doctoral mathematics program at the University of Chicago while still a teenager, and is taken seriously enough by senior mathematicians in the novel to be discussed as a genuine peer rather than a curiosity. She is also an accomplished violinist with expert knowledge of historic Cremonese instrument-making, and she possesses what the novel presents as a near-total recall of what she reads. By the metrics typically used to track underrepresentation—admission, credentialing, expert recognition—Alicia is a success case for visibility.

This recognition follows a documented institutional history rather than a single admission: the novel establishes that Alicia has been readmitted to Stella Maris on a voluntary basis for the third time, and her psychiatrist, Dr. Cohen, holds a dual appointment as a practicing physician and a professor at the University of Wisconsin—a figure who embodies institutional authority in both its clinical and academic forms. Her mathematical standing is corroborated externally as well: she is awarded a fellowship to work with the topologist Alexander Grothendieck at the Institut des Hautes Études Scientifiques, placing her within one of the most exclusive circles in twentieth-century mathematics. Her family is itself embedded in institutional scientific power—her father worked on the Manhattan Project—which makes her own exclusion from evidentiary authority within a comparable institutional setting more striking rather than less: recognition, in Alicia’s case, runs in the family, while credibility does not. Even within her family, recognition of her intellect was double-edged: her grandmother, who raised her after her mother’s death, is recalled as having been unsettled by the scope of her intelligence rather than reassured by it.

Alicia’s exclusion from evidentiary authority is best understood as intersectional rather than singular. Her class position and her family’s standing in institutional science, already visible in her father’s work on the Manhattan Project, are what open the door to the rooms she occupies: her early identification as gifted and her admission to a doctoral program while still a teenager depend on resources and connections not available to most people of her era. Her psychiatric diagnosis is what closes the door on her credibility once she is inside those rooms. Gender alone cannot account for this pattern, since her male relatives share her class position without facing a comparable discrediting mechanism. It is this combination of gender, psychiatric status, and institutional context—not any single axis—that produces her specific form of recognition without authority.

What she lacks is authority over the interpretation of her own testimony. She experiences recurring hallucinations, led by a recurring figure she calls the Thalidomide Kid and referred to collectively in the novel as her “cohorts,” and she carries an unresolved grief, compounded by an incestuous longing, for her brother Bobby, who she believes has died in an accident. Her psychiatrist treats her account of her own mental states, her perceptions, and even her mathematical intuitions as material to be filtered through a diagnostic frame—she has been diagnosed with a psychiatric disorder—before it can be treated as credible. She is not disbelieved because she is unseen; she is disbelieved despite being examined at length by exactly the institutional authorities positioned to grant credibility. The novel ends without resolving this asymmetry: Alicia dies by suicide shortly after the sessions the novel transcribes, a detail established in *The Passenger*, the companion novel to which *Stella Maris* is a coda.

The case is useful analytically for exactly the reasons tokenism and intersectionality theory jointly predict: Alicia is not under-recognized, and her exclusion is not reducible to gender alone. She is watched, examined, and discussed at length, by her family, her professors, and her psychiatrist alike. Her exclusion operates entirely at the level of authority—of whose account of a situation is permitted to count as knowledge, and under which combination of categories—rather than at the level of visibility (Cooper, 2022; Frye, 2025). This decoupling is difficult to observe directly in survey or policy data, where recognition and authority are rarely disaggregated by intersecting category; a single dense case can make the distinction, and its intersectional structure, legible in a way that supports, rather than substitutes for, the systematic study this debate calls for.

## Discussion

This article has proposed “recognition without authority” as a conceptual addition to the empowerment-versus-ritual debate in gender visibility scholarship, grounded in existing sociological theory (tokenism, non-performativity, intersectionality) rather than in literary analysis for its own sake. The illustrative case drawn from *Stella Maris* is offered as a heuristic, not as the article’s central claim: it demonstrates that recognition and authority can be fully decoupled even in cases of maximal visibility, a pattern tokenism theory predicts but which is rarely made vivid in policy-oriented writing on visibility days. This decoupling is not confined to fiction: Rossiter (1993) sociological concept of the “Matilda effect” documents an equivalent pattern in the history of science, in which women such as Rosalind Franklin, whose X-ray diffraction data was instrumental to the discovery of DNA’s double-helix structure, were fully visible within their institutions yet were denied the interpretive authority to have their own contributions credited on their own terms. The literary case examined here dramatizes, at the level of a single life, a decoupling that Rossiter’s sociological account documents across an entire scientific field. This also suggests that recognition without authority is not evenly distributed across identity categories. Future empirical work testing this concept should disaggregate recognition and authority along more than one axis, rather than treating gender as the sole variable of interest.

Future research could test this decoupling more systematically—for instance, comparing institutional recognition metrics (citations, appointments, media coverage) against measures of decision-making authority (grant review panels, editorial boards, promotion committees) for women in fields with active visibility campaigns, to establish whether “recognition without authority” is a general pattern or one specific to the kinds of institutional contexts a single literary case can illustrate but not generalize. The concept may also extend usefully to non-binary and gender-nonconforming subjects, whose relationship to existing recognition categories raises related but distinct questions beyond the scope of this article.

## Data Availability

The original contributions presented in the study are included in the article/supplementary material, further inquiries can be directed to the corresponding author.
